# Robot-assisted upper limb therapy for personalized rehabilitation in children with cerebral palsy: a systematic review

**DOI:** 10.3389/fneur.2024.1499249

**Published:** 2025-01-06

**Authors:** Daniela Cardone, David Perpetuini, Marta Di Nicola, Arcangelo Merla, Giovanni Morone, Irene Ciancarelli, Antimo Moretti, Francesca Gimigliano, Alice Cichelli, Francesco De Flaviis, Alex Martino Cinnera, Teresa Paolucci

**Affiliations:** ^1^Department of Engineering and Geology, University “G. d’Annunzio” of Chieti-Pescara, Pescara, Italy; ^2^Department of Oral Medical Science and Biotechnology, Physical and Rehabilitation Medicine, BIND, CARES, University “G. d’Annunzio” of Chieti-Pescara, Chieti, Italy; ^3^Department of Life, Health and Environmental Sciences, University of L’Aquila, L’Aquila, Italy; ^4^San Raffaele Institute of Sulmona, Sulmona, Italy; ^5^Department of Medical and Surgical Specialties and Dentistry, University of Campania Luigi Vanvitelli, Napels, Italy; ^6^Multidisciplinary Department of Medical and Surgical Specialties and Dentistry, University of Campania “Luigi Vanvitelli”, Naples, Italy; ^7^Department of Mental and Physical Health and Preventive Medicine, University of Campania “Luigi Vanvitelli”, Naples, Italy; ^8^Scientific Institute for Research Hospitalisation and Health Care IRCCS Santa Lucia Foundation, Rome, Italy

**Keywords:** rehabilitation, robotics, hemiplegia, upper extremities, exoskeleton, endeffector

## Abstract

**Introduction:**

Cerebral palsy (CP) is a group of permanent disorders of movement development that may cause activity limitations. In this context, robot-assisted therapy might play a key role in clinical management. This comprehensive systematic review aimed to investigate the efficacy of robotic systems in improving upper limb (UL) functions in children with CP.

**Methods:**

PubMed, EMBASE, Scopus, and PEDro were searched from inception to February 2024. The risk of bias was assessed with the Joanna Briggs Institute critical appraisal tools battery.

**Results:**

Of 756 articles identified, 14 studies involving 193 children with CP with a judged to be of good methodological quality, but with a lack in the study design, were included in the final synthesis. In the included studies a wide range of devices was used, both exoskeletons and end-effectors, both wearable and non-wearable. The CP children who underwent robot-assisted therapy reported a significant overall increase in clinical assessment, specifically in UL movements and manual dexterity. The clinical improvement was often accompanied by a gain also in instrumental assessments (i.e., kinematic analysis, EMG).

**Discussion:**

The present review suggested that robot-assisted therapy can improve UL motor functions in children with CP. Moreover, the availability of different devices with adjustable parameters can represent an important resource in proposing patient-centered-personalized rehabilitation protocols to enhance the efficacy of rehabilitation and integration into daily life. However, the limited sample size and lack of standardized and clearly reproducible protocols impose to recommend the use of robot-assisted therapy as an integration to usual rehabilitation and not as a replacement.

**Systematic review registration:**

https://osf.io/a78zb/.

## 1 Introduction

Cerebral palsy, which occurs in two to three out of 1,000 live births (1), has multiple etiologies resulting in brain injury that affects movement, posture, and balance. Movement disorders can be categorized as spasticity, dyskinesia, ataxia, or mixed, and spasticity is the most common movement disorder, occurring in 80% of CP (2). According to this evidence, CP is classified into spastic (80%), dyskinetic (15%), and ataxic (5%) forms. The focus of rehabilitation treatment (3–5) has recently shifted to neurological rehabilitation in response to increasing evidence for neuroplasticity. This approach aims to improve development and function by enhancing the capacity of the central nervous system to change and adapt throughout the patient’s life. As the life expectancy of CP children generally approaches that of the overall population, rehabilitative therapies must be developed with the aim to address the needs of children first and then adults aging with disability and provide a guide for developing shared goals with families considering the six “Fs” framework: function, family, fitness, fun, friends, and future (4, 6). Robotic systems are new devices that are becoming increasingly popular as a part of the treatment for CP (7) to improve gross motor function of the upper limbs (UL) and the fine motor function of the hand. Traditionally, based on the International Classification of Functioning, Disability, and Health, Children and Youth (ICF-CY) conceptual framework, constraint-induced movement therapy (mCIMT) and bimanual training (BIT) are the main physiotherapeutic techniques used in the recovery of UL function in children with CP (8, 9). Rehabilitation robots can provide “controlled, intensive task-specific training that is goal directed and cognitively engaging,” a concept consistent with the current emphasis on therapeutic interventions for UL function in children with CP in a successful association or alternation with traditional physiotherapy (7). Findings of recent research support robotic systems in combination with Virtual Reality (VR) settings to enhance UL rehabilitation in CP with the motivational features built into the interactive VR games (10). Robotic interfaces allow multiple methods to shape UL movement patterns (11, 12) and could represent a facilitation in the complex reaching movements with finalized game-like virtual simulations that can accomplish the repetition, attention, and ecological validity required for effective massed practice also for assisted play for functional and playful activities. The robotic system with virtual assistance has been clinically validated to be significantly more effective, compared to both unassisted and typical approaches, by increasing the hand controllability, reducing the physical load, and increasing the easiness of maintaining movements within the lines. Participating in a robot-mediated play activity has demonstrated to increase children’s motivation and, at the same time, engagement with the important resource for home-based implementation of the technology to promote manual play activities for children with disabilities (13). Robot-assisted training (RAT) provides a promising alternative; however, there is a need for solutions that specifically target children and their needs, especially on improving UL function and fine hand movement. However, the paucity of group design studies summons the need for more rigorous research before conclusive recommendations can be made (14, 15). Another important aspect of robot training is the mixed use of RAT with biofeedback systems. Biofeedback techniques play a significant role in enhancing motor skills through various mechanisms. Studies have shown that neurofeedback training (NFT) can effectively improve motor performance by allowing individuals to manipulate their brain activity with sensory feedback (16). Additionally, biofeedback relaxation training has been found to enhance athletes’ motor skills by improving the balance between sympathetic and parasympathetic nerves, increasing heart rate variability, and promoting psychological relaxation. Furthermore, multimodal biofeedback, involving EEG, ECG, PPG, and RSP signals, has demonstrated promising results in improving both motor and nonmotor symptoms in patients with Parkinson’s disease, highlighting the potential of biofeedback in enhancing motor functions through signal regulation (16). These findings collectively emphasize the valuable contribution of biofeedback techniques in optimizing motor skills and performance across different populations and contexts. Combining biofeedback with robotic training shows promise in enhancing UL rehabilitation for children with CP. Studies have highlighted the effectiveness of integrating biofeedback systems within wearable robotic devices to improve user engagement and muscle activity (17, 18). Additionally, research on RAT combined with transcranial direct current stimulation (tDCS) in children with CP suggests feasibility and safety, emphasizing the importance of careful participant selection and protocol adaptations for successful outcomes (19). These findings underscore the potential of feedback and biofeedback-enhanced RAT (20) to optimize UL rehabilitation outcomes in CP patients. This offers a novel approach to improving motor functionality and quality of life in this population. Therefore, the objective of this study was to review the effectiveness of robotic systems either as a therapy alone or in combination with the physiotherapy treatments of CP children in improving UL function as primary outcomes and their autonomy and quality of life as secondary ones.

## 2 Materials and methods

We conducted a comprehensive systematic review to explore the effect of RAT for upper limb recovery in children with CP. We summarized the results of all published studies following the Preferred Reporting Items for Systematic Reviews and Meta-Analyses (PRISMA) guidelines (21). The protocol was recorded in the Open Science Framework (OSF) register.^1^

### 2.1 PICO question

We used the PICO model (Population, Intervention, Comparison, Outcome) to define the search strategy and report the results (22). The PICO tool was allowed to respond to the research question: “Is robot-assisted therapy effective in promoting upper limb motor recovery in CP patients?” (the PICO strategy is reported in Table 1).

**Table 1:** PICO questions for search strategy.

PICO Elements	Keywords	Search terms	Search strategy
P (patients/population)	Children with cerebral palsy	Cerebral palsy	“Cerebral palsy” OR “Children” OR “Pediatric population” OR “Pediatric patients”
I (intervention)	Robot-assisted therapy	Robotics	“Robotics” OR “Robotic exoskeleton” OR “Exoskeleton device” OR “Robot-assisted therapy”
C (comparison)	Sham stimulation, usual care, no treatment	/	/
O (outcome)	Upper limb motor functions	Upper limb motor Recovery	“Upper limb motor impairment” OR “Upper limb motor rehabilitation” OR “Upper Limb” OR “Upper limb recovery” OR “Upper limb function”

### 2.2 Search strategy and eligibility criteria

A systematic search was conducted for all peer-reviewed articles published from inception to the first of January 2024, using the following databases: PubMed, EMBASE, Scopus, and PEDro. Mesh terms and free terms about the topic have been used: “cerebral palsy,” “robot-assisted therapy,” and “motor recovery” (the complete search strategy is reported in the Supplementary Appendix A). We included all studies involving children with CP subjected to RAT. Specifically, the inclusion criteria were: (i) children with CP (pediatric population (<18 years) affected by CP); (ii) RAT (alone or combined with other rehabilitation approaches); (iii) written in English language; and (iv) published in a peer-reviewed journal. We have excluded articles describing theoretical models, methodological approaches, algorithms, and basic technical descriptions. We excluded: (i) animal studies; (ii) conference proceedings and review; (iii) studies involving healthy children; (iv) single sessions investigations; and (v) feasibility studies. Considering the limited literature available, we included multiple study designs for qualitative synthesis: (i) Randomized Controlled Trial (RCT); (ii) Controlled trial; (iii) Longitudinal study (iv) case series; and (v) case study.

### 2.3 Data extraction and analysis

All search results were imported into an online database (RYYAN) (23) and screened by two blinded reviewers (F. D. and A. C.). In the first screening, duplicated articles were removed. After that, both reviewers screened the title and abstract to select eligible articles according to the inclusion/exclusion criteria. After screening based on titles and abstracts, the blind was opened, and the cases of disagreement were discussed with a third reviewer to allow a consensus (A. M. C.). After full-text selection, the data extraction from the included studies was reported on a sheet. Data from the fully read articles were extracted by the two reviewers (A. M. C. and A. B.). The population, age, type of robotic systems (intervention), outcome measures, and follow-ups were registered in a synoptic table. When there was any form of disagreement, a third reviewer was consulted (T. P.).

### 2.4 Risk of bias

The risk of bias of the individual studies was assessed with the Joanna Briggs Institute critical appraisal tools battery (24, 25). JBI is used to evaluate trustworthiness, relevance, and results via a specific tool for each study design and is useful in the case of comprehensive reviews with heterogeneous design (25–27). The risk of bias was assessed by two independent reviewers (A. B. and A. M. C.). Potential discrepancies in quality assessment were resolved through consensus or through discussion with a third reviewer (T. P.).

## 3 Results

**Figure 1:**
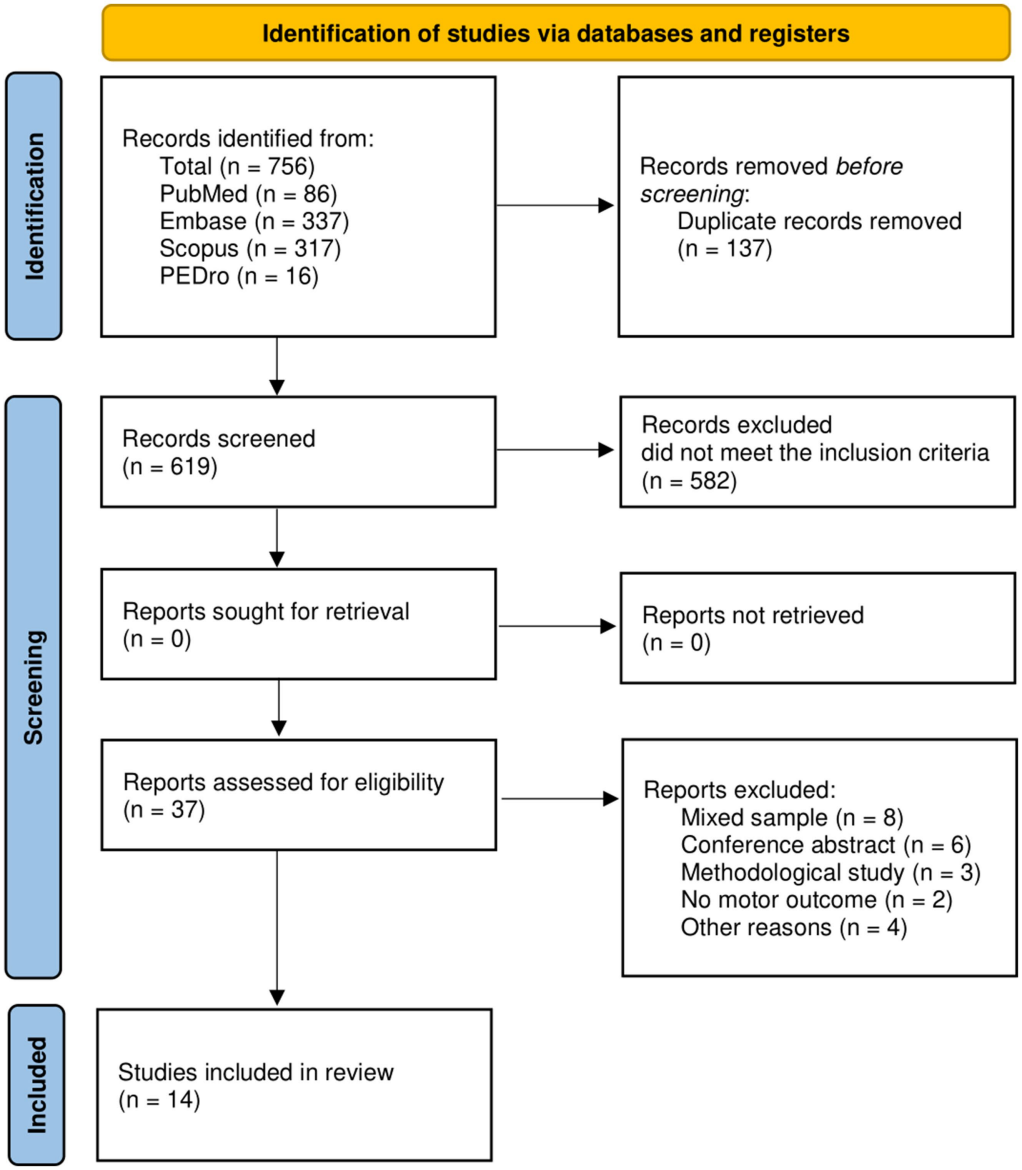
PRISMA Flow-diagram of studies selection. Source: Page et al. (63).

In total, 756 articles were found. After duplicate removal (137), 619 articles were screened by title and abstract and 582 were excluded because did not met the inclusion/exclusion criteria. Finally, 37 studies were extracted for the full-text analysis. Of the 37 full-text screened, 14 studies (10, 11, 28–39) were included in the synthesis (Figure 1). We found 2 RCTs, 9 longitudinal studies, 2 case series, and a single case. The included studies were published from 2008 (11) to 2022 (36). About 42.9% of studies were conducted in the USA, whereas 21.4% in Italy (see Table 2).

**Table 2:** Synoptic table of the included studies.

Author, year, country	Study design (Setting)	CP type (Classification)	Sample (*N* age ± SD gender)	Weeks (*N* sessions) [duration]	Device (Type)/Intervention	BFB (Yes = Y; No = N)	Outcomes	Results	Conclusions
Cimolin 2018, Italy	Longitudinal, Clinical	NR, MACS I-III	21 7–14 (range) NR	4 (40) [45]	Armeo® Spring (Exoskeleton)	Y	*Clinical:* QUEST, Melbourne *Instrumented:* *robot based Kinematics Assessment during reaching movements*	Clinical: ↑ QUEST ↑ Melbourne Instrumented: an increase was observed in speed.	RAT seems to be a promising intervention for improving UL function in hemiplegic children.
Colazza, 2021, Italy	Longitudinal, Clinical	NR, NR	5 9 ± 3 1 M, 4F	4 (20) [45′]	InMotion2 (End-effector)	Y	*Clinical:* BBT, NHPT, Melbourne, AHA *Instrumented:* Assessment by robotic system evaluation tools	*Clinical:* = BBT, = NHPT, = Melbourne = AHA *Instrumented:* an increase was observed in speed and smoothness.	The robotic training produced an increase in the activities of the hemiplegic.
El-Shamy, 2018, Egypt	RCT, Clinical	Spastic, MACS I-III	*Exp:* 15 6.9 ± 0.8 9 M, 6F *Ctrl:* 15 6.8 ± 0.8 11 M, 4F	12 (36) [45′]	*Exp:* Armeo® Spring (Exoskeleton) *Ctrl:* Passive stretching, weight-bearing exercises, stimulation of the protective reactions of upper limbs, bilateral hand use.	Y	*Clinical:* MAS, QUEST	*Clinical:* ↑ MAS, ↑ QUEST	RAT is significantly more effective than conventional therapy in improving the quality of UL movements in children with hemiplegic CP.
Fasoli, 2008, USA	Single case, NCR	NCR, NR	1 8.5 1F	8 (16) [60′]	InMotion2 (End-effector)	Y	*Clinical:* MAS, QUEST, FMA-UE Parent questionnaire *Instrumented:* Elbow strength	*Clinical:* = MAS, ↑ QUEST, ↑ FMA-UE = Parent questionnaire *Instrumented:* no minimal detectable change was observed	RT combined with BTX-A injections led to improvements in UL coordination and quality of motor performance in a child with moderate impairments attributable to CP.
Fluet, 2010, USA	Longitudinal, Clinical	NR, FLH	9 9.4 ± 3.7 5 M, 4F	3 (9) [60′]	NJIT-RAVR System (End-effector)	Y	*Clinical:* Melbourne *Instrumented:* Grip strength; AROM	*Clinical:* ↑ Melbourne *Instrumented:* an increase was observed in speed, smoothness, efficiency, and AROM flexion of shoulder (kinematics), and in the grip strength (force).	Three small pilots of NJIT-RAVR training demonstrated measurable benefit with no complications, warranting further examination.
Gilliaux, 2015, Belgium	RCT, (NCR)	NR, (MACS II-V)	*Exp:* 8 10.8 ± 4.6 NR *Ctrl:* 8 11 ± 3.5 NR	8 (16) [45′]	REA Plan (End-effector) *Ctrl:* Conventional Therapy (neurodevelopmental therapy).	Y	*Clinical:* BBT, QUEST, MAS *Instrumented:* Robot based Kinematics Assessment. The strength of 2 muscular groups (elbow flexors and extensors), assessed with a hand-held dynamometer.	*Clinical:* ↑ BBT, = QUEST, = MAS *Instrumented:* an increase was observed in speed, and smoothness.	RAT improved upper limb kinematics and manual dexterity but did not improve functional activities and social participation.
Keller, 2017, Switzerland	Longitudinal, Clinical	Spastic, Dystonic, Ataxic, mixed (MACS I-III)	11 13.3 ± 3.4 9 M, 2F	2 (3) [70′]	Armeo® Spring (Exoskeleton)	Y	*Clinical:* BBT, Melbourne *Instrumented:* *robot-based* Kinematic Assessment	*Clinical:* ↑ BBT, = Melbourne *Instrumented:* no greater effects were observed.	They found evidence indicating the successful acquisition, transfer, and retention of UL skills in children with CP subjected to RAT. Therefore, motor learning occurred when children with CP trained their more affected arm with weight-support in a playful, virtual environment.
Kolobe, 2019, USA	Longitudinal, Home	NR, NR	24 0.4–0.5 (range) 14 M, 9 F	12 (24) [15′]	Self-Initiated Prone Progression Crawler (SIPPC) robotic system (Other)	N	*Clinical:* MOCS *Instrumented:* SIPPC gathered robot and infant trunk/limb movement data	*Clinical:* ↑ MOCS* Instrumented: an increase was observed in the rotational amplitude, wrist path length, and linear path length.*	These findings suggest movement learning and retention in infants with CP is differentially affected using RL and EBL, with a combination of both showing more promise than RL alone. The findings also implicate cognition, type of brain insult, emergence of reaching, and muscle force production, which must be explored in future studies.
Krebs, 2012, USA	Longitudinal, Clinical	NCR, NR	12 5–12 (range) NR	8 (16) [60′]	InMotion2 (Exoskeleton)	Y	*Clinical:* FMA-UE, QUEST, MAS, Parent’s Questionnaire *Instrumented:* *robot-based* Kinematic Assessment	*Clinical:* ↑ FMA-UE, ↑ QUEST, ↑ MAS, ↑ Parent’s Questionnaire Instrumented: an increase was observed in movement duration, aim, deviation from the straight line, and smoothness.	Motor habilitation in CP exhibits some traits of motor learning. Optimal treatment may not require an extensive repertoire of tasks but rather a select set to promote generalization.
Kuo, 2020, Taiwan	Case series, Clinical	NR, NR	7 11 ± 4.5 6 M, 1F	6 (12) [40′]	Gloreha Sinfonia + dynamic support system (Exoskeleton)	Y	*Clinical:* FMA-UE, ABILHAND Kids, BBT *Instrumented:* UL-EMG of brachioradialis and extensor digitorum communis; Grip strength	*Clinical:* ↑ FMA-UE, = ABILHAND Kids, = BBT *Instrumented:* an increase was observed in the EMG activity of brachio-radialis muscle.	RT using a Gloreha device which focuses on the distal part of the UL benefit on body structure and function, including UL motor function, brachioradialis muscle recruitment, and coordination in children with CP.
Kuroda, 2022, Japan	Case series, Clinical	Spastic, MACS III-IV	3 15 ± 4.9 3 M	32 (9–13) [50′]	Hybrid Assistive Limb + Integrated Volitional Control Electrical Stimulation (Exoskeleton)	NR	*Clinical:* ARAT, QUEST, ABILHAND Kids, CHEQ 2.0	*Clinical:* ↑ ARAT, ↑ QUEST, = ABILHAND Kids, = CHEQ 2.0	The use of VAUT, together with new systems such as HAL and IVES, for severe CP is safe and may be effective. Our study suggested that UL function can be improved for patients with severe CP.
Peri, 2016, Italy	Longitudinal, Clinical	NR, NR	14 8–16 (range) NR	3–4 (15–20) [30′]	Armeo® Spring (Exoskeleton)	Y	*Clinical:* Melbourne *Instrumented:* *“P,” an index of the overall performance*	*Clinical:* ↑ Melbourne Instrumented: an overall improving trend was observed in the P index.	The parameter described here was able to show variations in performance over time and enabled a quantitative evaluation of motion abilities in a way that is reliable with respect to a well-known clinical scale.
Roberts, 2020, USA	Longitudinal, Clinical Camp	NR, MACS I-III	31 9.3 ± NR 16 M, 15F	1.5 (10) [30′]	Armeo® Spring (Exoskeleton)	Y	*Clinical:* AHA, Melbourne, COMP, Satisfaction.	*Clinical:* ↑ AHA, ↑ Melbourne, ↑ COMP, ↑ Satisfaction.	A P-CIMT camp augmented by the Armeo Spring Pediatric was feasible and accepted by participants. Bimanual hand function and occupational performance improved immediately following intervention, and the treatment effects persisted 6 months following intervention.
Qiu, 2011, USA	Longitudinal, NCR	NR, MACS II-IV	9 10.1 ± NR 8 M, 1F	3 (9) [60′]	NJIT-RAVR System (End-effector)	Y	*Clinical:* timed tests of: forward, sideways, hand to mouth reaching *Instrumented:* robot-based Kinematic Assessment	*Clinical:* timed tests of: ↑ forward, ↑ sideways, ↑ hand to mouth reaching *Instrumented:* an increase was observed in the path length, duration, and smoothness	No relevant conclusions.

AHA, assisting hand assessment; ARAT, action research arm test; AROM, active range of motion; BBT, box and block test; BFB, biofeedback; BTX-A, botulinum toxin type-A; CHEQ, children’s hands-use experience questionnaire; CG, control group; COMP, Canadian occupational performance measure; CP, cerebral palsy; EBL, error-based movement learning; EG, experimental group; F, female; FLH, functional level of hemiplegia; HAL, hybrid assistive limb; IVES, integrated volitional control electrical stimulation; M, male; MACS, manual ability classification system; MAS, modified Ashworth scale; MOCS, movement observation coding scheme; NCR, not clearly reported; NHPT, nine hole peg test; NR, not reported; P-CIMT, pediatric constraint induced movement therapy; QUEST, quality of upper extremity skills test; RL, reinforcement learning; RT, robot therapy; RAT, robot assisted therapy; RMT, robot mediated therapy; RPS, reaching performance scale; SIPPC, self-initiated prone progression Crawler; UL, upper limb; FMA-UE, Fugl-Meyer assessment scale for upper extremity; VAUT, voluntary-assisted upper limb training.* This data refers to children received RAT with reinforcement learning and error-based learning.

### 3.1 Population

The population of included studies involved a total of 214 children, 193 children with CP, aged between 4.5 months (33) to 16 years (36), and 21 healthy controls. In the ten studies that reported the gender (10, 11, 28–35), there were 87 males (64%) and 49 females (36%). Three studies reported the CP sub-types, two of these included children with spastic CP (30, 36) and the other one included all CP subtypes (33). Concerning the classification, eight studies reported the severity of UL impairment using the Manual Ability Classification System (MACS) (28, 30, 31, 33, 34, 36–38) or the Functional Level of Hemiplegia (FLH) (10). Only two studies (28, 35) reported a baseline cognitive assessment of total intelligence quotient and nonverbal intelligence, respectively.

### 3.2 Intervention

The included studies performed a RAT protocol of at least 3 sessions (32) to 40 sessions (28) with a mean of ~17 sessions. The duration of each session ranged from 15 min (36) to 70 min (32) with a mean of ~47 min. Four studies performed an hour of training (10, 11, 35, 39) and the other four performed 45 min of training (28–31). Regarding the total duration of RAT protocol, the range was between 10 days (36) to 32 weeks (36) with an average of ~7.6 weeks. The most recurrent total duration was 8 weeks (11, 30, 35, 39). Finally, the frequency (sessions/week) ranged from 0.25 (36) to 10 (28) with a mean of ~4 sessions per week. In seven studies, two or three sessions per week is the most adopted frequency (10, 11, 29, 35, 36, 38, 39).

#### 3.2.1 Devices characteristics and setting

**Figure 2:**
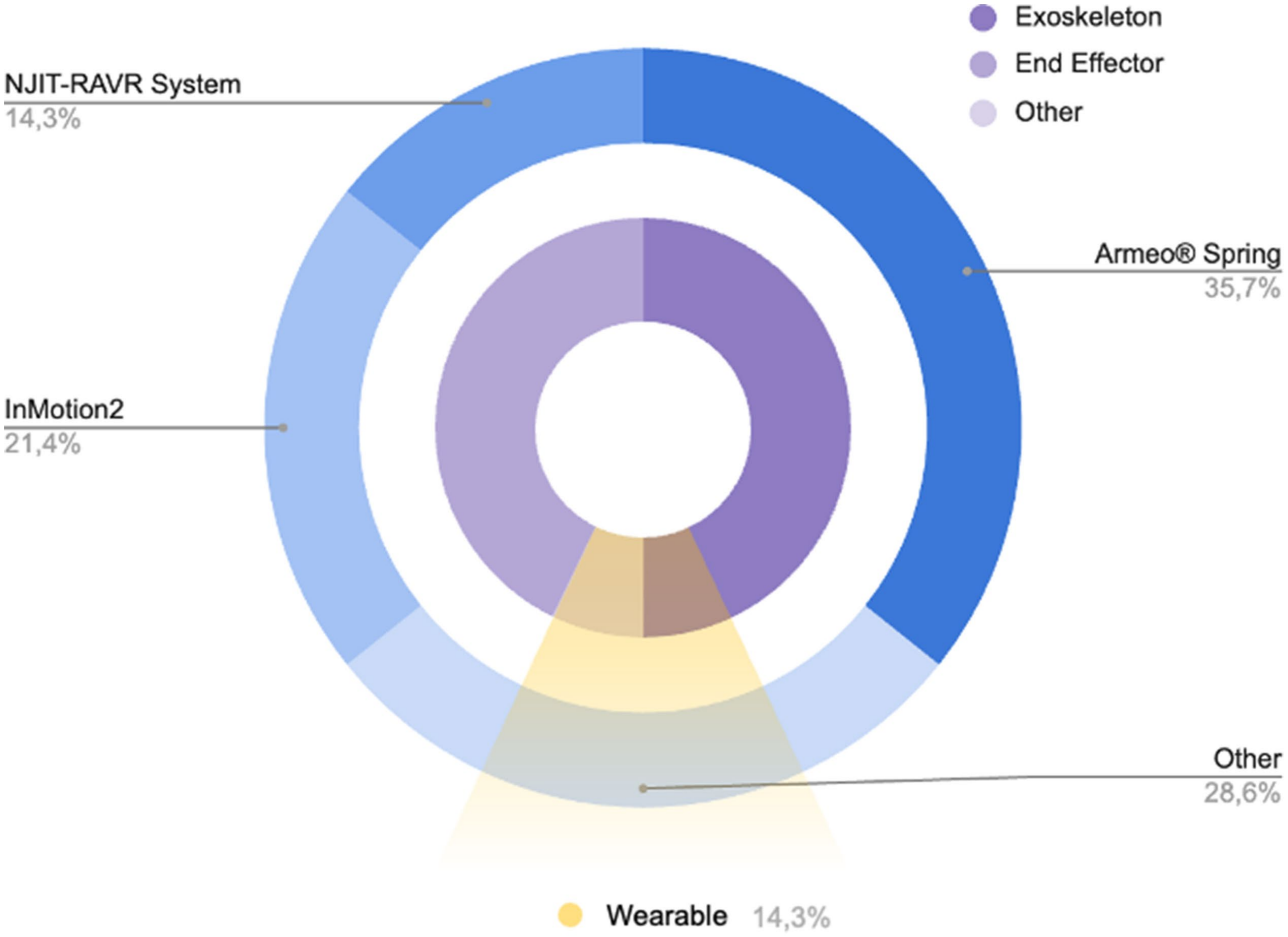
Pie chart showing the distribution of devices used in the included studies (blue pie chart) and their main characteristics (purple chart and yellow slice).

About the device used, in the included studies 50% used an exoskeleton device, 42.9% used an end-effector device and Kolobe et al. (33) used an integration of robotics and sensor technologies designed to capture and influence movement effort as infants learn prone locomotion. Specifically, five studies (28, 30, 32, 36, 37) used the Armeo® Spring device, three studies (11, 29, 35) used the InMotion2 device, two studies (10, 38) used the NJIT-RAVE System, and the other four studies (30, 31, 33, 36) used: the REAPlan; the Self-Initiated Prone Progression Crawler (SIPPC) device; The Gloreha Sinfonia with a dynamic support system; and a Hybrid Assistive Limb (HAL) with an Integrated Volitional Control Electrical Stimulation (Figure 2). Relatively to the settings, most of the studies were clinical (85.71%), and only two studies were nonclinical longitudinal (33, 39). Two studies used wearable devices (33) for flexion-extension of the elbow and for prone locomotion. However, only Kuroda et al. (36) used the wearable device in an environmental setting (home or childcare center). None of the cited works using wearable devices involved remote monitoring from home. Roberts et al. (38) and a subgroup of children (group III) of the study by Fluet et al. (10) performed RAT protocol in a clinical camp during a scheduled daily routine.

#### 3.2.2 Feedback and biofeedback

Twelve studies (10, 11, 28–36, 38) used an integrated feedback system via audio-visual and/or haptic stimuli often in the form of exergaming. Fluet et al. (10) used stereoscopic glasses to enhance depth perception, which increases the sense of immersion. Few studies used an immersive virtual reality environment during RAT. In the other two studies (33, 36) functional tasks were performed during RAT (reaching/grasping and prone locomotion).

#### 3.2.3 Combination with other therapies

In two studies (11, 29) the RAT protocol was administered after Botulinum Toxin Type-A injection (3 out of 5 children for (29)). In the study of Kuroda et al. (36) RAT therapy was coupled with online neuromuscular electrical stimulation. In two studies (10, 38) the RAT was administered inside a daily routine of other rehabilitative activities (i.e., CIMT and intensive bimanual therapeutic interventions) in a clinical camp. In one RCT study (30) the patients allocated in the experimental group underwent a mixed program of 40 sessions subdivided into 16 RAT sessions and 24 sessions of usual care. Other studies investigated exclusively the effect of RAT without other concomitant therapies.

### 3.3 Comparison

Only two studies compared the effect of RAT with respect to control groups subjected to conventional therapy (30, 31); another two (33, 36) compared the kinematics data of RAT in CP children with respect to healthy controls (15 and 6, respectively). Moreover, Kolobe et al. (33) split the CP sample into two RAT subgroups subjected to reinforcement (9 children) and reinforcement plus error-based learning (14 children). Fluet et al. (10) divided the children into 3 subgroups, the first two differing for the range of motion of prono-supination, the last one (4 children) performed the same number of sessions as group 1 and 2 (9 sessions) but with a frequency of 3 sessions per week plus 5 h daily of other rehabilitative activities (for more detail see Paragraph 3.2.3). Both RCTs (30, 31) performed the same dose of exercises, in terms of intensity, duration, and frequencies, in both experimental and control groups. El-Shamy (30) subjected the RAT group to 40 sessions of treatment composed of 16 sessions of RAT and 24 sessions of conventional therapy, while the control group performed 40 sessions of conventional therapy only. In the three studies (30, 31, 36), no declared statistical differences at the baseline were reported between groups except for Gilliaux et al. (31) and Kolobe et al. (33) which reported differences in the coefficient of variation straightness (6.9 and 3.7%, *p* = 0.03) and in the MOCS scores between the two CP subgroups (16.84 and 11.55, *p* = 0.02), respectively.

### 3.4 Outcome

All included studies used clinical scales and tests to assess the changes in UL functions after RAT. The most used clinical scales were: the Melbourne Assessment (MA) (10, 11, 32, 33, 36, 38), the Quality of Upper Extremity Skills Test (QUEST) (11, 31, 33–36), and the Fugl-Meyer Assessment scale for Upper Extremity (FMA-UE) (28, 33, 35). About clinical tests, the most used was the Box and Block test (BBT) (31, 33, 35). Ten studies (10, 28, 31–38)also reported instrumented evaluation including kinematics features, electromyography activity, or grip force results. Specifically, nine studies (10, 28, 31–36, 38) performed kinematics analysis, eight of these (10, 28, 32–36, 38) using robotics output collected on the same devices used for the training, and the other one (31) with an optoelectronic system with passive markers (SMART-DX, BTS, Milan, Italy) and a video system synchronized with the optoelectronic system (BTS, Milan, Italy). Kuo et al. (35) used a surface EMG (sEMG) on brachioradialis and extensor digitorum communis muscles during reaching tasks. Finally, four studies (10, 11, 28, 35) evaluated the force using a manual dynamometer for grip or elbow strength. Eleven studies (10, 28, 31–38) performed statistical analysis to interpret the results. For the rest, two studies (32, 37) reported descriptive statistics and the last one (10) used the minimal detectable change to discuss the RAT effects. Only two studies (28, 35) used the international classification of functioning (ICF) in the choice of outcome measurements.

#### 3.4.1 Change in clinical outcome

In the two RCTs included (30, 31) a statistically significant increase in the RAT group with respect to the control group was observed in manual dexterity, spasticity, movement patterns, and hand function. All other studies (10, 11, 28, 31–36, 38) reported an improvement in the within-group/within-subject analysis performed on clinical data except for one (33). Specifically, four studies (32–34, 38) reported an improvement in all clinical scales and tests, and the other seven studies (10, 11, 28, 31, 35–37) in at least one clinical outcome. About clinical tests, four studies administered the BBT (28, 31, 33, 35) but only two of these (31, 35) reported a significant improvement in manual dexterity.

#### 3.4.2 Change in instrumental outcome

In the eight studies that investigated UL kinematics (10, 28, 30, 31, 33–35, 38), seven studies (10, 28, 30, 31, 33, 35, 38) reported an overall improvement after RAT treatment in the kinematics features or in their derived indices. Most recurrent changes were observed in smoothness (10, 28, 30, 33, 38) and speed (10, 28, 30, 31). Kolobe et al. (33) reported an improvement in the rotational amplitude, but not in the wrist path length, foot path length, and linear path length. Regarding sEMG changes, Kuo et al. (35) observed a statistically significant improvement in the brachioradialis activity in the co-contraction ratio and in the electrical agonist–antagonist muscle ratio. Finally, in the force evaluation, only one study (10) out of four studies (10, 30, 31, 35) reported an improvement in grip strength after RAT treatment.

### 3.5 Risk of bias

Eight out of the fourteen included studies were evaluated to have a low risk of bias (10, 28, 31–35, 38). Five studies were judged to have a moderate risk of bias (11, 30, 31, 36, 37). Finally, one study was considered to have a high risk of bias (33). In the RCTs, the impact on the judgment was mainly attributable to the blinding items. In the other studies, the issues included consecutive inclusion, unclear patient history, and missing conflict of interest, which affected the overall judgment (Table 3).

**Table 3:** Risk of bias of the included studies with JBI critical appraisal tools.

Author, year	Study design	R	Allocation	Baseline comparability	Blind patients	Blind Therapists	Blind Assessors	Treatment comparability	Adequate Follow Up	ITT Analysis	Outcome comparability	Outcome reliability	Statistical Analysis	Overall Bias
Gilliaux, 2015	RCT	YES	YES	YES	NO	NO	YES	YES	NA	YES	YES	YES	YES	Moderate
El-Sh am y, 20 18	RCT	YES	YES	YES	NO	NO	YES	YES	NA	YES	YES	YES	YES	Moderate

Author, year	Study design	Clear inclusion criteria	Reliable condition measure	Condition’s identification	Consecutive inclusion	Complete inclusion	Demographics	Clear clinical information	Clear Outcomes or Follow-up	Clear site/clinic demographic informations	Appropriate Statistical Analysis	Overall Bias
Kuo, 2020	Case series	YES	YES	YES	Unclear	NO	YES	YES	YES	YES	YES	Moderate
Kuroda, 2022	Case series	YES	YES	YES	YES	YES	YES	YES	YES	YES	YES	Low

Author, year	Study design	Clear demographics	Patient’s history and timeline	Clear current clinical condition	Clear assessment	Clear Intervention procedure	Clear post intervention condition	Adverse events	Provide take-away lesson?	Overall Bias
Fasoli, 2008	Single Case	YES	NO	Unclear	YES	YES	YES	NO	YES	Moderate

Author, year	Study design	Philosophical perspective congruity	Objective congruity	Methods congruity	Representation and analysis of data congruity	Results interpretation congruity	Influence on the study declared	Conflicts of interest	Clear participants representation	Ethical approval	Adequate conclusion	Overall bias
Cimolin, 2018	Longitudinal	YES	YES	YES	YES	YES	NA	YES	YES	YES	YES	Low
Colazza, 2021	Longitudinal	YES	YES	YES	YES	YES	NA	NO	NO	NO	YES	High
Fluet, 2010	Longitudinal	YES	YES	YES	YES	YES	NA	YES	YES	YES	YES	Low
Keller, 2017	Longitudinal	YES	YES	YES	YES	YES	NA	YES	YES	YES	YES	Low
Kolobe, 2019	Longitudinal	YES	YES	YES	YES	YES	NA	YES	YES	YES	YES	Low
Krebs, 2012	Longitudinal	YES	YES	YES	YES	YES	NA	YES	YES	YES	YES	Low
Peri, 2016	Longitudinal	YES	YES	YES	YES	YES	NA	NO	YES	YES	YES	Moderate
Qiu, 2011	Longitudinal	YES	YES	YES	YES	YES	NA	YES	YES	YES	YES	Low
Roberts, 2020	Longitudinal	YES	YES	YES	YES	YES	NA	YES	YES	YES	YES	Low

ITT, intention to treat; NA, not applicable; R, randomization process; RCT, randomized controlled trial.

## 4 Discussion

In this review, the efficacy of robotic devices, either as standalone therapy or in conjunction with physiotherapy, in improving UL function in children diagnosed with cerebral palsy CP is examined. However, the results should be interpreted with caution due to the heterogeneity, and generalization is not possible because of the limited number of RCTs with adequate sample sizes, which affects the inferential statistics. Nevertheless, the available data can be valuable in summarizing the initial evidence on the use of RAT in the rehabilitation of UL function in children with CP. From the literature investigation, RAT has been proven to be potentially effective for rehabilitating UL. Its objectives include enhancing the amount and intensity of therapy while also standardizing the treatment process (40). This approach employs rigorous, repeated, interactive, and personalized exercises to enhance motor learning (41). This kind of therapy is administered through both end-effector and/or exoskeleton devices. End-effector robots engage with patients via a solitary point, whereas exoskeletons contain several contact points that closely mimic the anatomy of the human limb (42). The variation in contact points might potentially impact the way the devices interact with the patient’s limb throughout the rehabilitation process. Exoskeletons provide a more extensive and evenly distributed support to the upper limb during training compared to end-effector robots, thanks to their many contact points. Exoskeletons are defined by their wearable design, which closely imitates the shape of the human limb, providing more natural and instinctive contact during rehabilitation sessions (42). This design element has the potential to increase patient involvement and comfort during treatment, which might result in better results for CP patients undergoing upper limb rehabilitation. Conversely, end-effector robots, which interact via a single point, may provide benefits in terms of simplicity and user-friendliness during training sessions. The exact contact point may enable targeted workouts and meticulous control over certain motions, which might be advantageous for addressing specific rehabilitation objectives in people with CP. Ultimately, both end-effector and exoskeleton devices have distinct features and benefits in upper limb rehabilitation for CP patients. However, exoskeletons with multiple contact points and wearable designs may provide a more comprehensive and natural interaction experience, potentially resulting in improved rehabilitation outcomes. Conversely, end-effector robots provide focused and accurate manipulation of motions, addressing rehabilitation requirements. Importantly, it is possible to integrate robotic therapy administered through both end-effectors or exoskeleton with biofeedback, which is essential to increase patients’ motivation (43). Several examples of feedback and biofeedback have been implemented so far with the aim of making the rehabilitation process more effective. For instance, the trajectory data from robot motions during rehabilitation exercises is sent to a computer’s virtual scene, which aids in movement coordination and improves the rehabilitation process (44). Moreover, a study on the use of biofeedback-enhanced therapeutic exercise video games for young people with CP demonstrated that the implementation of biofeedback improved engagement by offering autonomy and varying feedback presentation (43). This approach increased the efficiency, effectiveness, and engagement of the therapy, highlighting the positive impact of biofeedback on patient involvement. Additionally, the use of EMG biofeedback has been suggested as a training tool to improve muscle activation and decrease spasticity in children with CP, leading to increased cooperation and compliance with treatment (45). By providing visual feedback through EMG biofeedback, children can actively participate in rehabilitation, promoting rapid recovery and enhancing engagement in the therapy process. Importantly, biofeedback has the capacity to enhance central nervous system (CNS) activity, thereby impacting a range of physiological responses. Research conducted by Stubberud et al. (46) has shown that EMG biofeedback may decrease the level of cortical excitability and influence the resonance and oscillations of crucial feedback loops in the CNS. Likewise, kinematic biofeedback therapies have been proposed to impact the activity of the CNS allowing patients to consciously manage hidden physiological reactions by making these responses more apparent (47). In addition, motion compensation control systems based on electromyography (EMG) are often designed to enhance the active involvement of patients in rehabilitation training, with the goal of improving results (48). These mechanisms offer visual cues for muscle activation, allowing patients to visualize and adjust their muscle activation patterns during therapy, which can enhance engagement and motivation in rehabilitation sessions (49).

Moreover, biofeedback treatments have been associated with the regulation of autonomic nervous system activity. Research has shown that biofeedback training may improve baroreflexes and increase heart rate variability, suggesting a direct impact on autonomic nervous system function via central processes (50). The impact of employing biofeedback has been proved not only in outpatient environments but also for training done at home (51). This training can make use of telemedicine and telerehabilitation methods. These evaluations are vital for tracking progress and customizing rehabilitation therapies with precision. Moreover, the use of virtual reality therapy in conjunction with biofeedback has shown efficacy in enhancing UL functionality in individuals diagnosed with CP, including children and young adults (52). This can lead to long-term enhancements in motor function and overall quality of life (53).

In summary, comparing the considered studies, all of them found evidence in the improvement of skills, such as dexterity, and motor abilities. One study (11) showed that RT combined with BTX-A injections led to improvements in UL coordination and quality of motor performance. Another study (38) focused also on the mid-term efficiency of RAT, showing that the treatment effects persisted 6 months following intervention. However, overall results consisted in better outcomes in terms of clinical scales.

### 4.1 Limitations

The review of the literature regarding the employment of robotic therapy for the treatment of upper limbs impairment in children with CP revealed significant heterogeneity in study design. Due to the limited number of available RCTs, to broaden the synthesis of results and enhance the robustness of the findings, the inclusion criteria were expanded from the initial protocol to cover a broader age range, from birth to 18 years (previously 4–18 years). The criteria were also modified to include a wider variety of study designs focused on robotic rehabilitation protocols for the upper limb, while excluding single-session studies, theoretical models, methodological approaches, algorithms, basic technical descriptions, conference proceedings, and reviews. This variability includes differences in the type and intensity of interventions, the duration and frequency of therapy sessions, and the metrics used to assess outcomes. Such heterogeneity poses a potential limitation, as it complicates the comparison and synthesis of results across studies. Additionally, we did not select a specific measure for the primary outcome but focused on overall functional improvement of the upper limb. The goal was to assess any positive changes in the functioning of the affected limb, without restricting the measure to a particular aspect such as strength, dexterity, or functional ability. This broader approach allowed us to capture a more comprehensive view of therapeutic effects across various domains of upper limb function. A notable concern is the lack of standardized dosage of therapy, which is further complicated by the variability in the severity of symptoms among patients. This inconsistency highlights the need for the development and implementation of standardized protocols to ensure more reliable and generalizable findings in future research. Besides, the method used to assess robotic therapy (RAT) performance is not uniquely defined. In the included studies, kinematic assessment was performed using the kinematics output of the robot device, and only one study used an external system such as pre-post measures. The use of the robot’s kinematic output for evaluating the change in upper limb (UL) motor gain is controversial (54, 55). Other approaches (i.e., inertial measurement units, optoelectronic systems, and stereophotogrammetry) can support the instrumented evaluation to objectify the results (56) both in clinical and home-based environments (57). Another significant limitation identified in the review is the limited sample size of the studies. Collectively, the studies have investigated fewer than 200 patients, which restricts the generalizability and robustness of the findings. Small sample sizes can lead to reduced statistical power, increasing the likelihood of Type II errors and limiting the ability to detect meaningful differences or effects. Furthermore, larger sample sizes are crucial for the application of advanced statistical analysis methods, including those based on artificial intelligence (AI). AI-based methods, such as machine learning algorithms, require extensive data to accurately identify patterns, make predictions, and generalize findings to broader populations (58). A larger dataset would enable the application of these sophisticated techniques, potentially leading to more precise and individualized therapeutic approaches. Therefore, future research should aim to include larger cohorts to enhance the reliability of results and fully leverage the potential of AI in analyzing and optimizing RAT for upper limb rehabilitation in patients with CP. Several studies employed mixed approaches, incorporating additional therapies alongside robotic interventions, thus limiting the ability to isolate and evaluate the effectiveness of RAT as a stand-alone treatment (8). This methodological choice complicates the interpretation of results, as the observed outcomes cannot be solely attributed to RAT. The concurrent use of various therapeutic modalities, such as conventional physical therapy, occupational therapy, or pharmacological treatments, introduces confounding variables that obscure the specific impact of robotic intervention. Consequently, the mixed-method approach reduces the clarity and precision in determining the efficacy of robotic therapy independently. To address this limitation, future research should prioritize the design of studies that evaluate RAT in isolation, ensuring a clearer understanding of its direct effects and therapeutic potential for upper limb rehabilitation in patients with CP. This will facilitate more accurate conclusions and inform clinical practice and guidelines more effectively.

Another limitation is the lack of flexibility and adaptability in existing UL exoskeleton rehabilitation robots, making it challenging to meet the diverse needs of patients with CP and address individualized rehabilitation requirements effectively (59). Furthermore, studies have indicated that patients undergoing RAT may not necessarily exhibit significant improvements in upper limb movement concerning daily living activities, suggesting a gap in translating the benefits of robotic therapy into functional improvements in real-world scenarios for individuals with CP (60). This discrepancy between the outcomes of RAT and the actual performance in daily activities highlights a limitation in the effectiveness of current biofeedback-enhanced robotic rehabilitation approaches. In this context, more in-depth research is needed to assess the association between individual differences in clinical functionality and/or genomics and the predisposition of patients to achieve better results with RAT. Of course, it is a false generalization that RAT is for all CP patients. For individuals with severe spasticity, contractures, or joint deformities, robotic therapy may be limited or require significant accommodations. Coexisting medical conditions (e.g., epilepsy, cardiovascular problems) or complications such as skin breakdown may sometimes limit the use of robotic devices. Diagnostic neuroimaging techniques (such as fNIRS, fMRI) could increase the knowledge of how robotic therapy can affect improvement, in parallel with a genetic analysis of the specific phenotype of each individual patient. This would be in the perspective of precision and individualized medicine that is highly desired recently.

Moreover, the complexity and cost associated with long-term hospitalization for the rehabilitation of patients with CP pose significant challenges, indicating a barrier to widespread adoption of biofeedback-integrated RAT in real-world clinical settings. The need for continuous monitoring, adjustment, and personalized feedback in RAT for CP patients may also present logistical challenges in terms of resource allocation (61) and clinical implementation (18). Clear determination of positive results on the functionality and abilities of patients with CP could be the basis for a huge investment of funds by the world health system on RAT. After more in-depth studies on a large cohort of patients with CP with RAT methodology, it will be necessary to draw up guidelines that allow the use of this therapy focusing only on patients who could potentially benefit significantly from it.

Finally, it is of fundamental importance to evaluate that general improvements in clinical assessments do not always translate directly into significant functional improvements in the daily life of rehabilitated patients (62). It should be emphasized that patients must be periodically stimulated and simultaneously monitored if the best results on their abilities and performances are to be obtained.

### 4.2 Future perspectives

Future research directions for the employment of robotic therapy in the treatment of CP patients should focus on several key areas to enhance efficacy and integration into daily life. Firstly, studies should aim to standardize therapy protocols, including dosage, duration, and frequency, to enable more consistent and comparable outcomes. Additionally, increasing sample sizes will allow for the application of advanced statistical methods and artificial intelligence to optimize treatment plans and personalize therapy based on individual patient profiles. Moreover, the integration of wearable devices that provide biofeedback (63) can be explored to extend the benefits of RAT beyond clinical settings. These devices, connected through the Internet of Things (IoT), can offer continuous monitoring and real-time feedback, empowering patients to practice therapeutic exercises in their daily routines and ensuring adherence to prescribed therapy. In addition, more studies are needed also to further investigate the combination of RAT with the Botulinum toxin type-A injection. Some studies included in the present review already combined the two therapies to reduce spasticity and parallelly improve motor outcome (64–66) but future investigations are however needed to confirm the effect in CP children. Furthermore, while standardizing protocols is essential for consistency, it is equally important to customize therapy to meet individual patient needs. Providing biofeedback information to robotic devices enable dynamic modulation of the robot’s behavior in real-time, tailored to the patient’s specific requirements. This approach ensures that the therapy is not only standardized but also adaptable, optimizing the therapeutic outcomes for each patient. Telemedicine can facilitate this personalized approach, allowing healthcare professionals to remotely supervise and adjust the therapy based on the data collected by wearable devices, ensuring that each patient receives the most effective and individualized care. Emphasizing these future directions will enhance the effectiveness, accessibility, and personalization of RAT for CP patients.

## 5 Conclusion

The results of the present review suggested that RAT can be used to improve UL functions in children with CP. Specifically in a considerable subset of the included studies, the use of RAT increased UL movements and manual dexterity such as recorded via clinical scales and tests. Moreover, in some studies the movement gain was coupled with a reduction of spasticity. The clinical improvement was also confirmed by the instrumented evaluation where an increase in many kinematics parameters was observed. Despite the heterogeneity of devices (i.e., exoskeleton or end-effectors) can represent a limitation for the synthesis about RAT efficacy, on the other hand this broad range of devices, and the possibility to modulate its parameters, can permitted a personalization of therapy according to children’s development and ability. This characteristic of RAT can be useful in children with cognitive and learning difficulties to correctly adapt the proposal aiming for a tailored rehabilitative approach. Finally, the RAT feedback provided in the form of exergaming seems to have increased participation and therapy satisfaction. However, the limited sample size, the heterogeneity of protocols (in terms of dose, duration and type of device), and the unsatisfactory study designs brings about the necessity of further randomized controlled trials to confirm our deductions. Considering strengths and limitations of RAT, to date, the use of RAT can be recommended as add-on to conventional rehabilitation to improve UL functions in children with CP, but never as a replacement of it.

## Data Availability

The original contributions presented in the study are included in the article/Supplementary material, further inquiries can be directed to the corresponding author/s.

## Footnotes

^1^ https://osf.io/a78zb/
