# Supplementary material for: Robot-assisted upper limb therapy for personalized rehabilitation in children with cerebral palsy: a systematic review
Source: Front Neurol. 2025 Jan 6;15:1499249. doi: 10.3389/fneur.2024.1499249 (PMC11743387; doi:10.3389/fneur.2024.1499249)
Supplement: Supplementary file 1 [file Data_Sheet_1.DOCX]

Supplementary Material

# Search strategy

**PubMed:** (("Robotics" OR "Robotic exoskeleton" OR "Exoskeleton device" OR "robot-assisted therapy") AND ("upper limb motor impairment" OR "upper limb motor rehabilitation" OR "Upper Limb" OR "Upper limb recovery" OR "upper limb function")) AND ("Cerebral palsy" OR "Children" OR "Pediatric population" OR "Pediatric patients")

**Embase:** ('robotics'/exp OR 'robotics' OR 'robotic exoskeleton'/exp OR 'robotic exoskeleton' OR 'exoskeleton device'/exp OR 'exoskeleton device' OR 'robot-assisted therapy') AND ('upper limb motor impairment' OR 'upper limb motor rehabilitation' OR 'upper limb'/exp OR 'upper limb' OR 'upper limb recovery' OR 'upper limb function'/exp OR 'upper limb function') AND ('cerebral palsy'/exp OR 'cerebral palsy' OR 'children'/exp OR 'children' OR 'pediatric population' OR 'pediatric patients')

**Scopus:** ( ( "Robotics" OR "Robotic exoskeleton" OR "Exoskeleton device" OR "robot-assisted therapy" ) ) AND ( ( "upper limb motor impairment" OR "upper limb motor rehabilitation" OR "Upper Limb" OR "Upper limb recovery" OR "upper limb function" ) ) AND ( ( "Cerebral palsy" OR "Children" OR "Pediatric population" OR "Pediatric patients" ) ) AND ( LIMIT-TO ( DOCTYPE , "ar" ) ) AND ( LIMIT-TO ( EXACTKEYWORD , "Cerebral Palsy" ) )

**PeDro:** “Robot” & “Cerebral Palsy”
